# Designing a behavioral intervention using the COM-B model and the theoretical domains framework to promote gas stove use in rural Guatemala: a formative research study

**DOI:** 10.1186/s12889-018-5138-x

**Published:** 2018-02-14

**Authors:** Lisa M. Thompson, Anaité Diaz-Artiga, John R. Weinstein, Margaret A. Handley

**Affiliations:** 1 University of California, San Francisco, San Francisco, California USA; 2 Nell Hodgson Woodruff School of Nursing, Emory University, 1520 Clifton Road, Suite 226, Atlanta, GA 30322 USA; 3 Center for Health Studies, Universidad del Valle de Guatemala, Guatemala, Guatemala; 4 School of Medicine, Boston University, Boston, MA USA

**Keywords:** Household air pollution, Liquefied petroleum gas stove, Behavior change wheel

## Abstract

**Background:**

Three billion people use solid cooking fuels, and 4 million people die from household air pollution annually. Shifting households to clean fuels, like liquefied petroleum gas (LPG), may protect health only if stoves are consistently used. Few studies have used an implementation science framework to systematically assess “de-implementation” of traditional stoves, and none have done so with pregnant women who are more likely to adopt new behaviors. We evaluated an introduced LPG stove coupled with a phased behavioral intervention to encourage exclusive gas stove use among pregnant women in rural Guatemala.

**Methods:**

We enrolled 50 women at < 20 weeks gestation in this prospective cohort study. All women received a free 3-burner LPG stove and ten tank refills. We conducted formative research using COM-B Model and Theoretical Domains Framework (TDF). This included thematic analysis of focus group findings and classes delivered to 25 pregnant women (Phase 1). In Phase 2, we complemented classes with a home-based tailored behavioral intervention with a different group of 25 pregnant women. We mapped 35 TDF constructs onto survey questions. To evaluate stove use, we placed temperature sensors on wood and gas stoves and estimated fraction of stove use three times during pregnancy and twice during the first month after infant birth.

**Results:**

Class attendance rates were above 92%. We discussed feasible ways to reduce HAP exposure, proper stove use, maintenance and safety. We addressed food preferences, ease of cooking and time savings through cooking demonstrations. In Phase 2, the COM-B framework revealed that other household members needed to be involved if the gas stove was to be consistently used. Social identity and empowerment were key in decisions about stove repairs and LPG tank refills. The seven intervention functions included training, education, persuasion, incentivization, modelling, enablement and environmental restructuring. Wood stove use dropped upon introduction of the gas stove from 6.4 h to 1.9 h.

**Conclusions:**

This is the first study using the COM-B Model to develop a behavioral intervention that promotes household-level sustained use of LPG stoves. This study lays the groundwork for a future LPG stove intervention trial coupled with a behavioral change intervention.

**Trial registration:**

NCT02812914, registered 3 June 2016, retrospectively registered.

## Background

### Household air pollution

Forty-one percent of the world’s population cook with solid fuels (e.g. wood, coal, crop residue, and animal dung) [1, 2]. Inefficient solid fuel stoves produce large amounts of particulate matter (PM) and carbon monoxide (CO), as well as a range of toxic, mutagenic or carcinogenic by-products [3–7]. In 2016, household air pollution (HAP) was the 8th leading risk factor for death globally, attributable to between 2.8 million [8] and 4 million deaths annually [9, 10]. HAP is associated with many chronic diseases such as ischemic heart disease, stroke, chronic obstructive pulmonary disease, cataracts and lung cancer [10, 11]. For children, the damaging effects of HAP are often severe, ranging from pneumonia, low birth weight, stillbirth and preterm birth in children [12–14]. The global burden of disease from HAP is concentrated in the poorest households in low-income countries. However, given scant evidence of relationship between HAP and low birth weight and preterm birth, these two health outcomes are not included in HAP-related global burden of disease estimates [15], despite their contribution to a large proportion of infant mortality [16]. It is therefore important to conduct studies that assess household air pollution reductions among pregnant women, and subsequent birth outcomes.

### Stove technologies exist, but there is a gap in understanding stove use behaviors

Chimney cookstoves that vent indoor air pollution outdoors or clean fuel stoves that use electricity, solar or liquefied petroleum gas (LPG) have the potential to improve health. However, little attention has been paid to how household members participate in decisions around cooking practices. Shifting households from using traditional stoves to a new LPG stove may improve indoor air quality only if the stove meets the demands of the household and is used consistently. Studies need to examine current stove practices, including the “entrenched practices” that make it difficult to “de-implement” [17], or abandon, the traditional stove. These “entrenched practices” may go beyond intervening with the primary cook, and may slip into the realm of other important decision-makers, such as  the cook’s mother-in-law or spouse. Resistance to de-implementation may also be influenced by poverty, lack of access to clean fuels, or discriminatory practices against the poor, including denying loans for new stove purchases. Similarly to de-implementation studies in the field of medicine [18], even the cleanest stove will not be used if social and structural forces are not acknowledged.

A well-known problem for health practitioners and researchers is addressing the “know-do gap” that lies between knowing something needs to be changed, and having the ability to make changes [19, 20]. This same gap exists in poor households relying on solid cooking fuels. Educating women about health risks from smoke exposure will do little to change their daily need for cooking under high exposure conditions. In these settings, women are constrained by fuel supply and cost, the need to prepare food in a certain way, or other household members who prefer to use the traditional stove instead of the gas stove [21].

To date, three randomized stove intervention trials in Malawi [22], Mexico [23, 24] and Guatemala [25] have shown that even the most fuel efficient, well-maintained solid fuel stoves have not succeeded in lowering HAP levels sufficiently to reduce infant pneumonia [26]. The reasons for this lack of translation into practice are numerous including:

- New stoves are introduced into homes without efforts to assess cooking tasks required of the new stove (e.g., new stove can’t perform all of the cooking tasks required of staple foods) or to overcome barriers (e.g., refilling LPG tank) [27, 28]
- Exposures to other sources of air pollution, such as outdoor fires to cook animal fodder, use of kerosene lamps for lighting, visits to neighboring homes where open cooking fires are used, trash burning near the home, and the continued use of the traditional stove [29, 30] dilute the impact of an introduced clean fuel stove, such as gas or electric stoves.
- ‘De-implementation’ efforts to discourage the use of the traditional stove and fuel sources, such as incentivizing families to remove the stove from the home, have not been well integrated into intervention studies.

Implementation science has developed tools and methods to understand and intervene upon the resistance to the successful adoption and use of a clean fuel stove and the pervasive exposure to additional sources of HAP. However, few studies in this field have used an implementation science framework to systematically assess and address the underlying barriers and enablers of this household risk factor that affects three billion people globally [31].

### Stove research in Guatemala

Since 2000, we have conducted studies to reduce HAP in rural Guatemala where over 90% of rural households cook with wood [1]. We recently began a study using an implementation science approach to develop an intervention that promotes the sustained use of an introduced LPG stove. We will first discuss the evolving stove research before presenting the current study.

During the RESPIRE randomized stove intervention study (2002–04), a well-maintained wood-fired chimney stove (referred to as the *plancha*) reduced kitchen CO levels by 90%, but only reduced maternal personal exposure by 61% and children’s personal exposure by 52%, compared to control homes with open cooking fires [32]. During the NACER study conducted in the same region (2012–13), 48-h personal PM_2.5_ exposures among women who used the *plancha* ranged from 130 μg/m^3^ (standard deviation, SD 65) during pregnancy to 63.8 μg/m^3^ (SD 17.3) during the neonatal period [33]. However, the chimney stove was not able to reduce absolute PM_2.5_ to levels needed to protect human health (World Health Organization interim target of 35 μg/m^3^ averaged over a 24-h period) [34]. Based on these and similar findings around the world, there has been a recent call for an acceleration of research that go beyond locally accepted “cleaner” solid fuel stoves [31, 35]. However, full adoption in low-resources settings of the “cleanest” cookstoves (e.g. LPG, biogas, ethanol, electricity or solar) has yet to be determined.

### Implementation science and new stove adoption

Theory-driven behavioral change interventions that are grounded in an understanding of the enablers and barriers to change, and that address these factors head-on, can begin to fill in the “know-do” or implementation gap. A review of 55 stove interventions in 20 countries found that most behavior change techniques were employed during the adoption of a clean cooking stove, when accepting to use a new stove becomes the essence of program success or failure [36]. To our knowledge, no studies to date have applied behavior change theory to influence the sustained use of clean cookstoves among pregnant women exposed to HAP. The COM-B Model is a comprehensive “behavior system” that provides the structure for assessing capabilities, opportunities and motivations, the fundamental conditions that must be understood at multiple levels, prior to the development of effective interventions. Capabilities are demonstrated by necessary skills and knowledge. Opportunities are the social or environmental factors that are external to an individual and allow change to occur. Motivation is guided by processes, either reflective or emotional, that direct behavior. The COM-B Model forms the hub of the Behavior Change Wheel (BCW) framework and nine intervention functions surround this hub. The nine intervention functions address the gaps in capabilities, opportunities and motivations that, when implemented correctly and consistently, will lead to sustained behavior changes [37, 38]. Related to the COM-B Model, the Theoretical Domains Framework (TDF) is a synthesis of 84 theoretical constructs of behavior change organized into 14 domains [39] . Each of the COM-B components maps onto several unique TDF domains [38].

The COM-B Model/BCW has been used to develop interventions for low-income cancer patients [40], stroke victims [41], preventative health checklists for children [42] and diabetes prevention for Latina women with gestational diabetes [43]. One recent study in Uganda used an opportunity-ability-motivation model [44] to identify the behavioral determinants that would lead to the purchase and use of a locally-produced gasifier stove [45]. Studies like this one, which characterizes formative research within a behavior change theory and an application framework similar to the COM-B/BCW, allow researchers to anticipate the bottlenecks before the introduction of the stove into the local market [45].

The overall purpose of the present study, referred to as NACER II (*nacer* means “to be born” in Spanish), is to implement and evaluate an introduced LPG stove coupled with a phased behavioral intervention to encourage use of the gas stove over traditional stoves among a group of 50 pregnant women in rural Guatemala. We focused on pregnant women because these young women may be the most amenable to behavior change messaging (to protect their unborn infants from harm) and because the fetus is susceptible to air pollutants, perhaps even more so in utero than during infancy [46, 47]. In this Guatemalan population, we have found that women do not receive preferential treatment that would reduce their exposure to cooking smoke during pregnancy. However, during the immediate post-partum period (and up to three weeks after birth when they observe the *dieta*, or are in repose), other women are tasked with cooking. Thus, our messages during pregnancy included other family members who cooked in the home. Here we describe the formative research to design and implement a behavioral intervention to promote the sustained and exclusive use of the gas stove using the COM-B framework, Theoretical Domains Framework (TDF) and BCW. This study is innovative in its approach to reducing exposure to HAP, which includes provision of a gas stove complemented by a tailored behavioral intervention and implemented by peer educators from local communities. We aim to assess the feasibility and acceptability of a tailored intervention that targets the entire family to be used in a future randomized, controlled trial.

## Methods

### Participants and setting

This prospective cohort study began in May 2016 and concluded in June 2017. Participants were screened for eligibility by fieldworkers during their prenatal visits at the Guatemalan Ministry of Health Clinic in the Western Highlands of Guatemala. Fifty women who met eligibility criteria, and who were < 20 weeks gestation confirmed by physician-administered ultrasound, were enrolled in the study. Inclusion criteria were: 18–45 years old; non-smoking; responsible for cooking meals in the home; use wood-fired open fire or deteriorated chimney-stove (e.g. 2 or more defects in stove leading to observable smoke seepage into kitchen); purchase wood fuel (if they gathered free wood, more likely to continue wood stove use); no plans to migrate in the next year; and have a cell phone that we can contact (86% of households in the area have a cell phone). Exclusion criteria were: use gas stove as their primary stove in the home; multigestational pregnancy, associated with preterm birth and low birth weight; and, because women wore personal air pollution monitoring equipment during pregnancy, we excluded women with a child < 2 years routinely carried on mother’s back. After women met initial eligibility criteria, we visited them in their homes to assess the condition and types of cookstoves, to assure that their biomass stove was in poor condition and that they did not have a gas stove in the home. At that visit, women who agreed to participate signed a written consent. All women were visited at < 20 weeks, 24–28 weeks, 32–36 weeks, within 48 h of birth and when infant reached 1 month age. At three months, we visited the households to conduct a final survey about stove use at which time they were disenrolled from the study.

### Ethics approval and consent to participate

This study was approved by the Committee for Human Research at the University of California, San Francisco and the Ethics Committee at Universidad del Valle de Guatemala. All women provided written consent to participate after consents were verbally read to them in their preferred language, either Mam or Spanish. If women could not sign, they provided a thumbprint and the consent was witnessed by a third party.

### Study procedures

#### Focus groups

Prior to the NACER II study, we conducted focus group discussions to explore knowledge and behavior gaps in cooking and heating practices, as well as experience with LPG stoves. We used data from these focus groups to inform content in Phase 1 and 2 and are therefore described here. Briefly, a convenience sample of 34 recently pregnant women with children < 1 year of age was recruited to participate in four focus group discussions. These women had previously participated in a study to evaluate a wood-fueled chimney stove from the same communities in the rural Western Highlands. Three focus groups consisted of women using *planchas* (*n* = 24), five of whom also owned a LPG stove. One focus group consisted of women using open fires for cooking (*n* = 10). The open-ended questionnaire focused on: (1) choice of stove and fuel type; (2) factors that influence preferences for cooking stove or fuel type; (3) effect that cooking smoke has on family health; (4) methods to avoid smoke in the home; (5) positive or negative aspects to changing cooking stoves and fuels; and (6) potential home modifications to reduce smoke exposures.

A trained fieldworker of Mam-Mayan descent moderated the focus groups in Spanish and Mam. A second fieldworker, also of Mam-Mayan descent, took notes. Two additional Spanish-speaking field staff and the principal investigator (LT) were present for the focus group sessions. Each focus group lasted 45 to 60 min, and women received a small food gift for their participation. After the focus groups, researchers and fieldworkers discussed what they had observed and heard from participants. All focus groups were audio-recorded and transcribed into Spanish by the researchers. Atlas-ti (version 8.0.33) was used for data analysis.

#### NACER II study

In **Phase 1** of the NACER II study, 25 pregnant women received a free 3-burner LPG stove and ten free 25-pound tanks of gas, delivered during the course of the study until the child reached one month of age. Women participated in three interactive group classes using contextually appropriate course content based on focus group results. Two Mam-Mayan fieldworkers conducted the series of interactive classes in Spanish and Mam. Each class lasted 2 h and occurred every month for 3 months during pregnancy.

In **Phase 2** of the NACER II study we used the same procedures described in Phase 1 but we assessed a more resource-intensive behavioral intervention approach with a different group of 25 pregnant women. In this phase, we intensified our focus on household-level behavioral change, assessing relevant behaviors at the household level and planning activities to involve family members in the LPG adoption process. During the provision of the 10 free LPG tank refills, the air pollution engineer, the stove technician, and the Mam-Mayan fieldworkers visited each home to work with household members to identify barriers and enablers for LPG use and prioritize tailored behavioral strategies, which could realistically be made by household members. These tailored strategies were then discussed by the team, refined using the COM-B framework, and reassessed at subsequent household visits.

#### Stove use monitoring

We placed iButton® (Maxim Integrated) temperature data loggers on the gas stove as well as on any wood stove, indoors or outdoors, to measure usage changes when the new stove was introduced. Two iButtons were placed on the lateral sides of the gas stove to detect any gas burner in use. These sensors log temperature every minute, and show temperature rise and fall with stove use [48–50]. Multiple sensors on the gas stove provide an on/off signal, but do not allow us to tell how many burners are on during a single cooking event. We inspected the gas stove during household visits to ensure proper use.

#### Data analysis

For the focus group data, qualitative data analysis was conducted using thematic analysis as describe by Braun and Clarke (2006) [51]. We analyzed focus group transcripts to identify salient semantic themes using a deductive and inductive approach. The principal investigator (LT) and a research assistant coded the data independently and jointly resolved discrepancies in the coded data. Deductive themes were extracted from the data based on prior HAP research in this area of rural Guatemala. New themes expressed by participants were inductively identified and coded in vivo by both coders. From this, we created a list of 11 categories to target for the behavioral intervention. Both coders reviewed new themes that emerged, discussed theme discrepancies and developed a final list of themes. We mapped the TDF domains onto these themes.

During NACER II, we designed group interactive classes based on themes that emerged from the focus group discussions. In Phase 2, we used the COM-B Model to assess behaviors around stove use and designed intervention functions for a behavioral change intervention, as has been done in previous studies [43, 52].

From iButton sensors left in a location to measure ambient air temperature, we generated mean daily temperature and determined that the stove was in use if stove instantaneous temperature reading was above mean daily temperature. We summed total time each stove was in use during the 1-week monitoring period and standardized to *hours in use per day* for comparison across different gas and wood stoves and open fires. We compared median hours of stove use using the non-parametric Wilcoxon rank sum test between baseline at < 20 weeks gestation and subsequent rounds with gas stove (24–28, 32–36 weeks gestation and 24 h and 28–31 days postnatal).

## Results

### Thematic analysis of focus group discussions

**Fig. 1:**
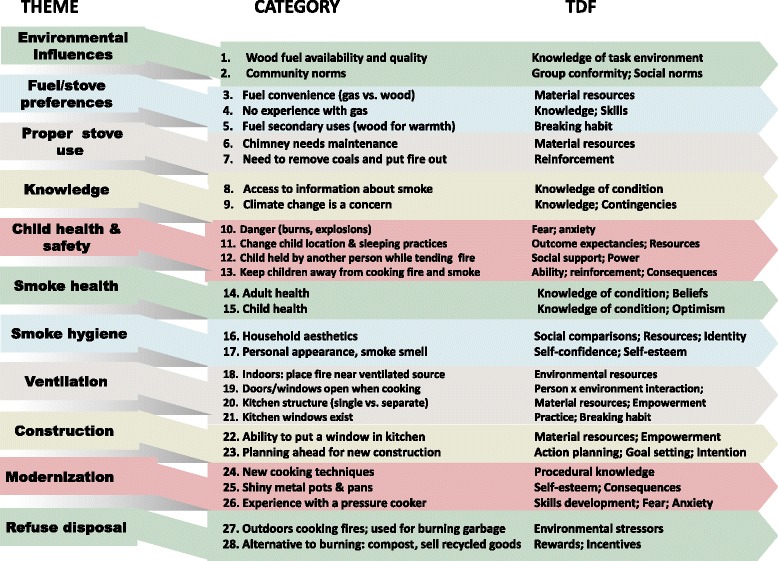
Eleven categories and 28 themes from thematic analysis of focus group discussions and theoretical domain framework [39] constructs

Focus group findings were summarized into 28 themes organized into 11 categories (Fig. 1) each with a critical role needed to support LPG stove use. Emerging themes were then categorized into whether they would be a barrier or would enable LPG stove use (Table 1). The first theme described was *environmental influences*, which included 1) wood fuel quality/accessibility, and 2) the social context for undertaking a cooking activity considered outside the community norm. For the first, wood can either be gathered freely on owned land, or can be purchased from delivery trucks that pass by homes. Participants viewed wood supply as abundant and accessible. However, wet or “green” (uncured) wood is very smoky and “bad to use”; during the rainy season, some women indicated that they have no choice but to use wet wood. For the second, participants described not wanting to appear different from their neighbors, to avoid envy or shame. Acquiring household goods, like a new gas stove, is desired, but discussing these purchases with neighbors was viewed negatively. One woman said, “We are all the same”.

**Table 1:** Enablers and barriers to liquefied petroleum gas (LPG) use

	Enabler	Barrier	Enabler or barrier
Free gathered wood		X	
Affordable wood delivered to home		X	
Shame of being different than neighbors if own new stove		X	
Worried neighbors will envy them if own new stove		X	
*Plancha* stove meets most cooking demands		X	
*Plancha* heats the room, providing warmth		X	
Older women are accustomed to using the *plancha* and afraid of gas		X	
Older women are afraid of gas		X	
Inorganic refuse needs to be burned in outdoor open fires		X	
Animal food needs long cooking times over open fire to not “waste gas”		X	
Difficult to access gas tank refills		X	
Explosion danger from gas stove		X	
Newly married women are financially constrained		X	
Younger women are not the decision-makers in the household		X	
Uncured or wet wood during rainy season is smoky	X		
During cold weather windows are closed, increasing smoke	X		
Burn danger to child if near open fire	X		
Knowledge about climate change	X		
Knowledge about health impacts of breathing smoke	X		
Clean kitchens, cooking pots and clothes	X		
Young women want to be “modern” and use gas stoves	X		
Cost of wood versus gas fuel is unknown			X
Men provide money for stove repair costs			X
Men are in charge of constructing doors/windows/kitchens			X
Smoldering fire is bothersome, but people learn to adapt			X

The second category was *fuel and stove preferences*. All women who owned a *plancha* stated that they prefer the *plancha* because “you can cook everything on it”. Women in the open-fire focus group were younger and newly married. They aspired to own a clean stove, but were living in their mother-in-laws’ homes, and could not yet afford to buy a stove. Women cook together in the home, and stated that older women are accustomed to using the open fire or the *plancha* and that it would be hard for them to learn to use an LPG stove. Only five participants with *plancha* stoves also had LPG stoves; they discussed the difficulty with LPG tank availability. LPG fuel tanks were delivered to households, but more commonly were picked up from a distributor in town. Women did not know whether wood or LPG gas was more costly. In these high-altitude communities of Guatemala, women felt that the *plancha* kept the house warm while the gas stove provided no additional warmth.

*Proper stove use and maintenance* was the third category. Having a chimney in good repair was important, but women stated that repairing or replacing the chimney required cooperation from their husbands, who would need to provide money for repairs. Women mentioned that smoldering fires produced smoke. One woman discussed blowing through a plastic hose to stoke the fire in the *plancha*; this kept her from leaning into the fire. In response, another woman joked, “we don’t do that, even the baby learns to blow on the fire”. The fourth category was general *knowledge* about HAP. This concerned issues of air pollution and climate change. One woman stated, “Burning trash, like plastic, heats up our environment and makes the sun hotter”. Another stated “we have heard about this on television”.

*Child health and safety* included many themes, but the most predominant themes were danger from burns when a child is near an open fire or danger from explosions when gas stoves or fuel tanks are improperly used. None of the women had experienced this, but had heard stories from other women about these potential dangers. To keep children safe, women mentioned keeping children outside the kitchen or asking other family members to care for children. Several younger women said they didn’t have support from others, and had to carry their children on their backs while they cooked. *Health related to smoke exposure* was the sixth category. One woman mentioned that a doctor told her that her child’s pneumonia was “caused” by smoke; another stated “doctors and nurses tell us smoke is bad for babies.” Women stated that breathing smoke “affects the lungs and eyes, especially in older women”.

The seventh category, *hygiene related to smoke exposure*, included a discussion of kitchens with clean instead of sooty walls, pots that were shiny and not blackened, and clothes that did not smell smoky, which made some women feel shameful. *Ventilation*, the eighth category, was possible if doors and windows were kept open, especially during the start-up of the fire when smoke was thick. Some women stated that elder family members preferred to keep the windows shut during cold weather; women did not feel that they could question those decisions. Stove placement was important. If the open fire was placed in a well-ventilated area, near a window, smoke would be minimized. Others stated that too much wind circulating in the kitchen made it difficult to control the gas flame. The ninth category was *construction*. Most women stated that men’s decisions around spending household income limited them from making changes they preferred, like adding a window to the kitchen. The women in the open fire group were thinking about new kitchen construction, but were unable to do so because they had not yet saved enough money to purchase materials and still lived with their mother in law. *Modernization* was the tenth category. Women spoke of the “old days” when women used clay pots on open fires, and about their current desire to use metal pots that stayed shiny because they use clean stoves. The final category was *refuse disposal*. Outdoor trash fires were burned to dispose of garbage, such as rubber shoes and plastic containers, because there was no garbage service. Open fires were used to cook animal food, and women mentioned burning plastic in these fires because they had no other way of disposing of ubiquitous plastic bags. Several women composted organic material and sold plastic bottles, but most were resigned to burning the inorganic solids that accumulate in their homes.

### NACER II study

During NACER II, 238 women were approached at the antenatal clinic by trained, Mam-speaking fieldworkers. Of those, 73 met the eligibility criteria and were < 20 weeks confirmed by ultrasound. Of the 73, two did not wish to participate in the study, two had miscarriages before they consented to participate in the study, six had chimney stoves in good repair and three already had a gas stove at the stove inspection visit, and ten could not be located for the stove inspection visit.

Among the 50 women who participated in the study, the mean gestational age at baseline was 13.1 weeks (range, 6.7–19.7 weeks) and for 14% of the women this was their first pregnancy (Table 2). Twenty-seven (56%) and 23 (46%) were recruited in the first and second trimester, respectively. The majority (86%) self-identified as Mam-indigenous. None of the households had a stove in the same room where they sleep; 88% percent had a stove in a separate structure from the main house. Ten (20%) used an open fire and 40 (80%) used a *plancha* stove without a chimney or in a deteriorated condition as their principal stove. Additional sources of smoke were as follows: 36 (72%) used a wood-fired steam bath (a *temascal*, or *chuj*) for bathing [53], 33 (66%) burned garbage near the home, 23 (46%) cooked animal food outdoors over an open fire and 7 (14%) were exposed to second-hand smoke (median of 1 cigarette/week smoked at home).

**Table 2:** Demographic and household characteristics of participants

Maternal characteristics	
Maternal age, years, mean (range)	25.4 (18.4–38.8)
Maternal education, n (%) (*n* = 49)	
None	13 (27)
Completed elementary school	13 (27)
Completed middle school	7 (14)
Completed high school	16 (32)
Principal source of income in the home	
Tenant farmers/works the land of others	25 (50)
Employee in public/private sector	13 (26)
Construction	4 (8)
Small business owner	3 (6)
Cultivates own land; weaves; sells animals	3 (6)
Maternal ethnicity, *n* (%)	
Mam-speaking, or indigenous	42 (86)
Spanish-speaking, or ladino	3 (6)
Both	4 (8)
First pregnancy, *n* (%)	7 (14)
Prior pregnancies, mean (range)	3.4 (2–10)
Gestational age at baseline, weeks (mean, range)	13.1 (6.7–19.7)
Household characteristics	
Socioeconomic status	
Crowding^a^, mean (range))	4.9 (1.3–12)
Economic support^b^, mean (range)	2.3 (1–6)
Dependency ratio^c^, mean (range)	3.8 (1.4–10)
Access to latrine, *n* (%)	49 (98)
Main water source during dry season, *n* (%)	
Piped water to home	33 (66)
Well water	9 (18)
Natural well (spring)	8 (16)
Main house structure materials, *n* (%)	
*Roof*	
Aluminum	44 (88)
Tile/Cement	6 (12)
*Walls*	
Adobe	29 (58)
Cinderblock/brick	16 (32)
Wood/Other	7 (14)
*Floor*	
Cement	28 (56)
Dirt	21 (42)
Tile	1 (2)
Exposure to air pollution	
Primary lighting source, *n* (%)	
Candles	2 (4)
Electricity	48 (96)
Primary heating source, *n* (%)	
None	43 (86)
Open fire	2 (4)
Wood chimney stove	5 (10)
Primary stove type	
Open fire	10 (20)
*Plancha* stove (no chimney or deteriorated)	40 (80)
Stove quality^d^	
Fair	4 (8)
Moderate	35 (70)
Poor	11 (22)
Cooking fuel type, n (%)	
Wood only	7 (14)
Wood and crop residue	22 (44)
Wood and plastic	2 (4)
Wood, crop residue and plastic	19 (38)
Traditional steam bath	
Usage per week, median minutes (IQR)	60 (30, 67)
Symptoms associated to cooking, *n* (%)	
Eyes irritated	42 (84)
Throat irritated/Causes coughing	35 (70)
Headache	30 (60)
Household decision-making	
*Large purchase items*	
Participant	2 (4)
Spouse	22 (44)
Both participant and spouse together	8 (16)
In-laws	12 (24)
Other family member	6 (12)
*Small daily purchases*	
Participant	6 (12)
Spouse	7 (14)
Both participant and spouse together	19 (38)
In-laws	12 (24)
Other family member	6 (12)
*Visiting relative*s	
Participant	5 (10)
Spouse only	17 (34)
Both participant and spouse together	23 (46)
In-laws	4 (8)
Other family members	1 (2)

^a^Crowding represents the ratio of the number of household inhabitants to the number of rooms in the house

^b^Number of adults providing economic support in the household

^c^Dependency ratio is the ratio of the number of household inhabitants to the number of adults providing economic support

^d^Stove quality cut offs are based on the total possible number of problems observed with the chimney stove during stove evaluation. The lower 25% quartile was considered to be fair, 25–75% moderate, and 75% and above poor quality. Open fires were categorized as poor quality

### Phase 1 and Phase 2 activities

Twelve to thirteen women attended each class in the 3-part series. Attendance rate was 93% for Phase 1 and 92% for Phase 2. Classes were based on focus group themes (Table 3). For example, we discussed feasible ways to reduce exposure to HAP since households often continued to use wood stoves. Nine behaviors were discussed, based on the evidence for HAP sources, and from the focus group findings: 1) cook in well-ventilated areas where air can circulate freely; 2) take turns cooking with other women in the family; 3) have other people care for the baby or children; let older children play outside during cooking; 4) find ways to prepare food that require less cooking time (while still thoroughly cooking food); 5) use dry wood, not wet wood, for cooking; 6) DO NOT burn plastic or garbage inside the kitchen; 7) make your fire at a distance no greater than 1.5 m from the closest window so that smoke can exit through the window; 8) keep windows and door open while the fire is burning; 8) use a fan or blow through a tube to fuel the fire; and 9) use LPG stoves that produce less smoke. These behaviors were reviewed at each class. Because we heard from women that stove safety was a concern, we reviewed gas stove and fuel tank safety, including how to detect and respond to a gas leak. We conducted one cooking event at each session: 1) a porridge drink (*atol*) was cooked at the first session to demonstrate ease of use, and how to cook a liquefied with a regulated flame to keep it from boiling over; 2) a “blind taste test” of beans cooked on an LPG stove and a *plancha* was conducted at the second session, with discussion about preference for one or another; and 3) a “cook off” of *atol* on an LPG stove and over an open fire was done at the final session, with a discussion of time savings, taste and appearance of the drink, and what women would do with time freed up from gathering wood, building fire and cooking for longer periods of time. Finally, at the last class, participants broke into three groups and acted out a socio-drama. They negotiated with “family members” (other participants) about refilling the LPG tank. This activity helped them anticipate discussions they would have at home after the study ended and fuel refills were no longer free.

**Table 3:** Objectives and learning strategies of classes offered in Phase 1 and Phase 2

	Objectives	Learning strategies
Class 1	Discuss “simple or easy” ways of preventing exposure to smoke from burning wood and other solid fuels	Photos used to generate discussion. Images include proper ventilation, well-maintained stove, children close to open fire
	Demonstrate use of gas cookstoves and discuss benefits and barriers to use	Facilitator cooks *atol* (corn-based beverage) over gas stove. Stove technician reinforces proper stove use and maintenance. Participants return-demonstrate lighting and cleaning stove
Class 2	Discuss options that exist for the reduction in exposure to smoke produced by burning wood to cook, in women and children	Women each receive photo representing 9 strategies to reduce smoke exposure. When moderator reads the strategy out loud, the woman with the photo attaches the photo to the wall
		Women work in groups to form puzzles with strategies to reduce smoke in the home
	Discuss maternal and child illnesses associated with exposure to smoke	Group discussion of personal/known experiences with illnesses such as pneumonia and low birth weight and relationship to HAP
	Discuss frequently mentioned barrier “food tastes better cooked on wood” compared to gas stoves	Blind taste test of beans prepared in identical manner, on wood and gas stove. Discussion of which food is better, and why
Class 3	Investigate perceptions regarding financial savings or costs related to gas stove that influence its use	Open discussion using flip chart to tally responses
	Discuss decision-making processes in the home that influences the sustained use of gas stoves and fuel	Small group role play “*we are out of gas, what do we do now?*”, followed by discussion of decision making in the home and how to improve communication
	Compare differences in cooking time when using wood versus gas fuels and discuss time savings/leisure time	Cook off: half of group cooks *atol* over an open wood fire, the other half cooks over a gas stove. Compare time saved, taste of food, and discuss how women might spend time if not using wood fires.

During home visits, the stove technician provided LPG tank refills and stove maintenance and reinforced messages around checking for gas leaks and keeping the stove clean and in good repair. We provided all homes with a calendar that illustrated safety messages about the gas stove. We used the calendar to keep track of delivered LPG tanks. At each visit, we discussed usage patterns, especially if the interval between tank visits was longer or shorter than expected.

### Intensive behavioral intervention approach in phase 2: The diagnosis

In Phase 2, we applied the COM-B model [37, 38] and the 14 related domains in the TDF [39]. Of the 84 TDF constructs, we mapped 35 constructs in 14 domains to questions we developed about behaviors related to LPG stove use. The remaining components were not relevant to the behavioral diagnosis. For example, professional roles and identity were not relevant for women who work at home. Punishment or sanctions, part of reinforcement, were not socially acceptable.

We attempted to be as specific as possible, to frame questions around women’s behaviors and, for the most part, to use the household as the target location (Table 4). We asked questions during home visits when LPG tank refills were delivered, usually every 2–3 weeks. At each visit a stove technician assessed the Capability component questions, and a bilingual Mam-speaking female fieldworker asked Motivation and Opportunity component questions. All members of the team assessed the household environment and family engagement at each visit. Because the list of target questions was considerable, the field team assessed up to four constructs at each visit until all domains had been assessed in the 25 homes. Home visits averaged 20 min.

**Table 4:** COM-B and Theoretical Domains Framework applied to women’s use of liquefied petroleum gas (LPG) stoves

Theoretical Domains Framework		
Domains	Constructs	General assessment questions for women in target households
Capability-Physical (COM-B)		
Skills	Skills Skills development	- Can women light the flame using a match and can they regulate the flame? - Can women connect the tank to the stove? - Can women turn gas on and off at the tank valve? - Can women detect and respond to a gas leak? - Can women lift and transport the tanks? - Can women keep children out of the way of the tanks when cooking or when not cooking?
Capability-Psychological (COM-B)		
Knowledge	Procedural knowledge	- Do women know the sequence of steps for use of the stove on a daily basis? - Do women know how to determine the tank is empty and how to turn stove off and attach a new tank? - Do women know how to get the tank refilled and delivered to their home?
	Knowledge (related to gas stove benefits)	Household Benefits - Do women know that using the gas stove makes a cleaner kitchen? - Do women understand the economic and time-saving aspects of the stoves? Health Benefits - Do women understand the health benefits of avoiding wood smoke for them and their children? Community or Ecological Benefits - Do women know that using a gas stove reduces deforestation/saves ecologic resources? Concerns - Do women have concerns about the stove, (e.g. based on collective myth about explosion risk or other concerns)?
	Knowledge of task environment	- Do women have the interpersonal skills to promote gas stove use within their families?
Memory, attention, and decision processes	Attention	- Can women remember how to use stove and tank, and can they demonstrate the steps involved?
	Decision making	- Can women recall a time when they were trying to cook and something happened to trigger them to stop using the gas stove and switch to the wood stove?
	Cognitive overload	- Can women concentrate on using the gas stove in different types of situations (e.g. when children are under foot, when mother in law is directing in the kitchen, when guests are present)?
Behavioral regulation	Self-monitoring	- Do women have any symptoms that prompt them to avoid smoke exposures (headache, eye irritation, cough)?
	Action planning	- Are there some unavoidable situations where women must use wood? What are they? - Can women plan ahead to use gas for parties with large amounts of food? - Can women plan ahead to cook the animal food early on when the tank is full?
Motivation-Reflective (COM-B)		
Social/Professional Identity	Identity (within household)	- How do women feel being the owner of the gas stove? How has the stove changed how they spend their day? - Does using the gas stove resonate with view of self as good cook and wife? - What have other family members said about the stove?
	Social identity	- Do women know others who use a gas stove? - Have women seen anyone else cooking with a gas stove outside their family? - How is the women perceived by friends and family perceive her using the gas stove?
	Group identity	- Is there a community group that women could work together with to promote gas stove use? - Would being part of a group of women who were using gas stoves enhance women’s roles in the community? - Would this strengthen women’s view of themselves?
Beliefs about capability	Self-confidence	- Do women feel confident they can use the gas stove for cooking most meals? Can women avoid using wood for cooking meals? Can women avoid using wood for other tasks?
	Perceived behavioral control	- Can women describe a time that they were not able to use the stove because others did not want them to? How confident were women about continuing to use the gas stove when this happens? - What things do women do to influence the health of their family?
	Self-esteem Empowerment	- Do women believe that their actions are important for changing their family’s health? - What things do women do to influence the health of their family? - How do women think that using a gas stove can help their family’s health?
Optimism	Optimism Pessimism	- Do women think that their actions can positively affect their children’s health? - Do women think that no matter what they do, they cannot make a difference in children’s health? In their health?
	Identity	- Do women see themselves in charge of children and the cooking realm in the home?
Beliefs about consequences	Beliefs	- Do women believe that changing to gas stoves will reduce the risk of illness to their children? To self? To others?
	Outcome expectations	- Do women believe that illnesses can be controlled/avoided by their own actions?
	Consequences	- What do women think will happen if they do not use the gas stove? - What will happen if women start using the gas stove? If they don’t like the gas stove, will they be able to change back to the wood stove easily? - What will happen if women stop using a wood stove? - What do women gain or lose from switching stoves? - What will happen if the woman tells their family that they are switching to a gas stove?
Intentions	Stability of intentions	- What are women’s intentions for purchasing wood in the future? - What are women’s intentions for purchasing refillable gas tanks in the future? - What are women’s intentions for repairing the gas stove if it breaks in the future?
Goals	Goals	- What are women’s goals for preventing respiratory illnesses in the family? - What are women’s goals for using a gas stove? Using a wood stove? - How much do they want to use a gas stove?
Motivation-Automatic (COM-B)		
Reinforcement	Rewards	- Are there any good reasons to use the gas stove over the wood stove? What are those reasons?
	Consequences	- Do women think that using the stove change has had other impacts in their home? Good or bad?
Emotion	Fear	- How afraid are women when they use the gas stove? - What makes women afraid of the stove? (probe on valve, gas leak, lighting, explosion, playing with knobs) - How afraid are women about their children getting too close to the gas stove? - What can women do to overcome these fears? - What are women afraid may happen if they use it? - Are others in the home afraid of the stove? What are they afraid of? - Do women have fears about the wood stove? What are those fears?
	Positive/negative affect	- How happy or proud are women about using the stove? - Have women’s interest in using this stove increased, or decreased or stayed the same over time?
Opportunity- Physical (COM-B)		
Environmental context and resources	Environmental stressors	- During certain seasons, is it harder to use a gas or a wood stove? What might make it easier to use the gas stove? - Have price fluctuations in the cost of tank refills affected the household? How?
	Resources/material resources	- How does the family procure gas tank refills? - Who does the stove repairs in the house? In the community? - What if the stove broke? What would the woman do? Would she be able to afford to repair it? - If she had to buy a new stove would she be able to do so? Would she have to borrow money? Can she buy a new stove on credit from a store?
	Barriers and facilitators	- Is it hard to get gas tank refills? Why? Discuss how she would go about refilling the tank when it is empty - Is it hard to make repairs to the gas stove? Why? Discuss a time when she had to repair her stove and what she did
Opportunity- Social (COM-B)		
Social influences	Social pressure	- Do family/friends pressure women to use either the wood or the gas stove? Why does she think that happens? What if women don’t agree to use the wood stove? Can she describe an instance where this happened?
	Social supports	- Do husbands provide support for tank refills and stove repairs? How? What kind of support? - Do other friends and family provide support? How? What kind of support?
	Group norms	- Do women think that other women similar to them are starting to use gas stoves? - Do women see themselves as setting an example for other women by using a gas stove? How does that make women feel?

We addressed constructs in the Capability component of COM-B (specifically skills, skills development and procedural knowledge), such as showing household members how to regulate the gas flame so that heat could instantly speed up or slow down cooking. However, the construct of fear (in the Automatic Motivation COM-B component) was related to skill development, because women had “heard stories” from family and neighbors that made them hesitant to use gas stoves. Reflective Motivation constructs, such as identity, empowerment and intentions were challenging to elicit and often involved ongoing interactions with female fieldworkers, who could speak with women about their own experiences with gas stoves.

Young women, often living in households belonging to their in-laws or parents, expressed their inability to make household changes, despite their intentions to use the LPG stove; this affected many of the motivational and opportunity domains. One woman stated, “If my mother-in-law tells me to light the *plancha* because she is cold, I have to obey”. One the other hand, one woman said, “Now even my dad cooks with the gas stove”. Women who did not live with their in-laws were more likely to state positive intentions to use the gas stove. One woman stated that she was proud to own her stove, and when she moved to her own home, she would take the gas stove with her. In terms of group norms, a component of the Social Opportunity construct, women didn’t know other women who had prior experience with LPG stoves. One woman stated, “I am a good example to my neighbors. I am happy because my house is free of smoke and that keeps me healthy. But I feel bad because some women may think I am *too full of pride* now that I own a gas stove”.

### Intensive behavioral intervention approach in phase 2: The intervention functions

We developed several intervention functions from the BCW based on focus group results, group interactions during Phase 1 classes and the diagnostic questions applied in 25 homes [38]. We tailored household-level interventions to extend the messages from the class setting to the home. We addressed issues that related to the capability factors in that environment. A set of proposed intervention functions guided each tank refill visit (Table 5). To illustrate, in Phase 1, we trained women to use the stove, but we found that mother-in-laws were resistant. During the post-partum period, when women were in repose during the first three weeks, older women cooked for the family and returned to using their biomass stoves. Therefore, in Phase 2, we trained other cooks in the home to light and regulate the flame at the tank refill visits (Training function). Because women’s spouses make decisions about large purchases, including stove repairs, tank refills and home construction, in Phase 2 we involved spouses in discussion of what can be done to add a window to the kitchen where the biomass stove is located to ventilate the smoke (Environmental Restructuring function). Despite apparent enthusiasm for the LPG stove, lack of money was the primary reason for inability to make home modifications or repair stoves.

**Table 5:** Examples of Intervention Functions implemented by fieldworkers to promote LPG gas use behaviors among women who are the household cooks

Behavior Targeted	Intervention function	Example	Method for assessing whether intervention function was achieved
Initiation of stove use and safety checking	Training	With another cook in home (preferably mother-in-law), demonstrate sequence of lighting stove, changing fuel tank and checking for leaks	At next visit, other cook return-demonstrates steps for one of these sequences.
Recognize and avoid smoke exposures in family members, including children	Education	With other adult family members in home, discuss signs and symptoms of low birth weight, pneumonia and chronic obstructive pulmonary disease. Explain why smoke from cooking fires exacerbates conditions. Ask if they have seen conditions and what they can do to avoid smoke.	At next visit, household member states two health conditions or diseases caused or exacerbated by HAP and names one action they have done to avoid smoke exposure.
Cooking with gas stove instead of woodstove	Persuasion	Show video of several women cooking together in smoke-free clean kitchen, with testimony from mother-in-law about how she likes her kitchen clean and free of smoke. Ask if this reflects the reality in their home.	At next visit, praise them if they have used their gas stove more frequently (measured by days from last tank refill). If they have not, ask them what they remembered about video they saw last time.
Cooking with gas stove instead of woodstove	Incentivization	Tell family that they will receive a prize if they use their stove two times a day for one week.	At next visit, reward the family with small bag of beans and rice if they adhered to stove use for 1 week
Cooking with gas stove instead of woodstove saves time	Modelling	Fieldworker discusses her use of gas stove and how this has benefitted her daughters-in-law who live with her, including their ability to earn more income performing other tasks, like raising animals to sell.	Ask woman if she remembers the conversation with the fieldworker from the previous visit. Ask her what things she could do with more time freed up from cooking.
Initiate tank refill before field team’s next visit (family level)	Enablement	Ask tank delivery service to visit home with fieldworker to provide contact information and discuss future deliveries.	Within one month after end of study, family has elicited LPG tank refill and the tank was delivered to the home.
Attempted/successful trouble shooting discussion with others in home of kitchen smoke risks (family level)	Environmental Restructuring	Discuss material costs for modifying kitchen to include an extra window with husband. Show a video of a man discussing the benefits to his family after he made this change. Trouble-shoot barriers and enablers to making structural changes in the home.	At the end of the study, reassess if any household modifications have been done that improves ventilation in the kitchen, such as new window or added door, or new kitchen where gas stove is located.

In our future intervention work, video vignettes presented on an electronic tablet in the home will be used to stimulate household-level conversation about the proposed changes will be developed. An example of wood stove dis-implementation could show a baby on a mother’s back while she is building the fire, and a sister-in-law who offers to carry the baby on her back outside of the kitchen while the woman is building the fire. An example of gas stove implementation would be a video showing beans cooked in traditional clay pots over a gas stove. These modelling exercises could stimulate a conversation among all household members about the possibility of doing this in their home in a non-judgmental way.

**Fig. 2:**
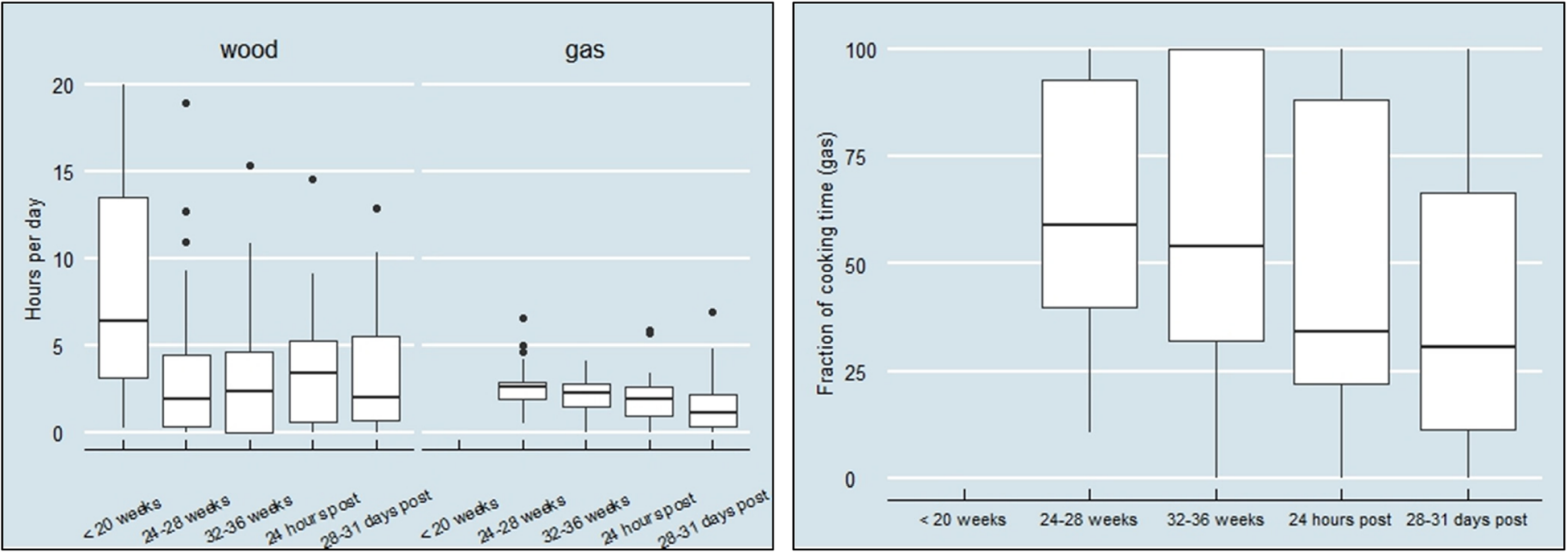
Stove use monitoring of wood and gas stoves during pregnancy and post-partum periods, by hours in use (left panel) and fraction of gas stove use (right panel)

Wood stove use dropped upon introduction of the gas stove from 6.4 h cooking duration, or 1.8 events at baseline to 1.9 h cooking duration, or 1.0 event at 24–28 weeks gestation (Fig. 2). The number of cooking hours decreased significantly across all rounds compared to baseline (Wilcoxon rank sum; *p* < 0.001). However, wood stove use increased gradually over the course of the study, with a concomitant decrease in gas stove use from 2.6 h, or 3.8 events at 24–28 weeks gestation to 1.1 h, or 2.1 events at 1 month post-partum (Wilcoxon rank sum; p < 0.001). We saw a marked decrease in the fraction of cooking represented by gas stove use from nearly 60% after introduction (24–28 weeks gestation), to 54% (32–36 weeks gestation), to just over 30% (at 1 month post-partum).

## Discussion

This is the first study to examine the use of the COM-B Model to develop a behavioral intervention that promotes household-level use of LPG gas among young, pregnant women. To date, engineering solutions have focused on “building the best stove” but years of research have shown that efficient, clean-burning stoves are inconsistently used when behaviors are not reinforced, broader environmental factors not incorporated, and family-level dynamics are not directly brought into the intervention design strategy. Understanding and intervening on the cook’s behaviors within the social setting of household cooking is a new approach, and studies are starting to develop an implementation science approach in this manner. This approach would likely improve the sustained use of LPG stoves. A quote attributed to Albert Einstein states, “If I had an hour to solve a problem I'd spend 55 minutes thinking about the problem and 5 minutes thinking about solutions”. Thus, strategies to change behavior should not be “decided upon” without a deep understanding of the complex behaviors that are either barriers or facilitators of change, regardless of our enthusiasm to ‘do something’.

Because household decisions are frequently made by other more powerful decision-makers in the home, rather than the woman who does most of the cooking, young women desiring to shift to an LPG stove may not feel empowered to do so. Once they have the stove, refilling the LPG tanks needs to be consistent. In our study, large household purchases were made by spouses (44%) or the mothers-in-law (24%). It is critical to encourage and reinforce gas stove and fuel use among other important household decision-makers. Interventions likely will need to be more complex, involving more household members, and over longer periods of time. This was reflected in the stove use monitoring data, which demonstrated higher use of the LPG stove during pregnancy than during the post-partum period when other members of the family made decisions to resume use of the wood stove.

Reflective motivation constructs, such as identity, beliefs, intentions and goals were difficult to elicit because participants found them to be abstract. These deep-rooted thoughts and emotions can either motivate or limit the impact of an intervention. Even when a trained female fieldworker facilitated the class in women’s preferred language, some women were not able to “speak their mind” in this setting. Deference to authority is a strong social norm in this part of rural Guatemala. This is seen in the homes, where women defer to their in-laws, and in public, where women defer to clinicians and educators. In this milieu, understanding or engendering “empowerment” is challenging.

Given that the LPG stove was a new technology, we emphasized the sustainable use of the gas stove, including how to negotiate LPG tank refills during the classes. Class activities were well-received by women, 27% of whom had no formal education. For example, the socio-drama was instrumental in helping women act out the steps required to negotiate the purchase of a LPG fuel tank with their household members. The local LPG distributor accompanied us at the final tank delivery and explained the process for future tank delivery. Two to three months after the households received the last free tank, we visited participants to see if they were able to purchase LPG fuel, which at the time of this study costs less than wood fuel. Fifteen households (34%) bought wood and 22 (51%) bought gas after the last tank was given to them. Of those who did not buy gas, the three most commonly cited were lack of money (8, 38%), haven’t finished the gas provided by the study (7, 33%), and family members didn’t want to purchase (2, 10%). None of the households stated that they couldn’t find a distributor or that it was difficult to exchange cylinders. Early adopters of a new technology are often seen as people who can positively motivate change in a community. Among young women in our study, being seen as different from their neighbors was thought to cause envy. In an effort to avoid this, women did not want to broadcast their satisfaction with the LPG stove. Thus, a diffusion of innovations approach [54] may move slower in communities that value group conformity or may need to be modified to incorporate other household members to also promote adoption, such as husbands who make decisions about fuel purchases and mother in laws who share cooking responsibilities in the home.

### Limitations

There were several limitations to this study. First, this was a formative research study with 50 participants. We have provided here several intervention functions that we piloted, and which may serve as examples for others. Second, this study was located in the Highlands of Western Guatemala where several high pollution exposure patterns predominate: the use of the *plancha* indoors when weather is cold; the use of the wood-fired sauna bath (*temascal*) for bathing; and weekly use of an outdoor open fire to cook animal food for many hours. Thus, even if women use the gas stove consistently, these other contributors to HAP will persist.

## Conclusions

Despite global efforts to develop wood stoves that are more efficient and “cleaner”, these stove do not consistently reduced HAP to levels known to protect human health. LPG stoves can reduce HAP when fully adopted [55]; however, the sustained use of these stoves is challenging. We described the first study to use the COM-B Model/TDF/BCW to deliver a behavioral intervention that promotes household-level use of gas stoves, the cleanest intervention available for many who live without reliable electricity. The use of this model will lay the groundwork for a future LPG stove intervention trial coupled with a behavioral change intervention. Given complicated learning processes around using a LPG stove and tank, behavioral interventions should be developed based on local contexts. The conditions in Guatemala will be different from the conditions in other countries. By using the COM-B Model/TDF/BCW method described here to tailor intervention functions to local contexts, future studies may achieve the desired change to use LPG stoves exclusively, and ultimately improve the health of women and children globally.

## Abbreviations

BCW: Behaviour Change Wheel
CO: Carbon monoxide
HAP: Household air pollution
LPG: Liquefied petroleum gas
PM_2.5_: Particulate matter, 2.5 μm in diameter
TDF: Theoretical Domains Framework
